# Supplementary material for: Inferring direction of associations between histone modifications using a neural processes-based framework
Source: iScience. 2022 Dec 7;26(1):105756. doi: 10.1016/j.isci.2022.105756 (PMC9813700; doi:10.1016/j.isci.2022.105756)
Supplement: Document S1. Figures S1–S9 and Tables S1–S3 [file mmc1.pdf]

iScience, Volume *26*

Supplemental information

Inferring direction of associations between histone modifications using a neural processes-based framework

Ananthakrishnan Ganesan, Denis Dermadi, Laurynas Kalesinskas, Michele Donato, Rosalie Sowers, Paul J. Utz, and Purvesh Khatri

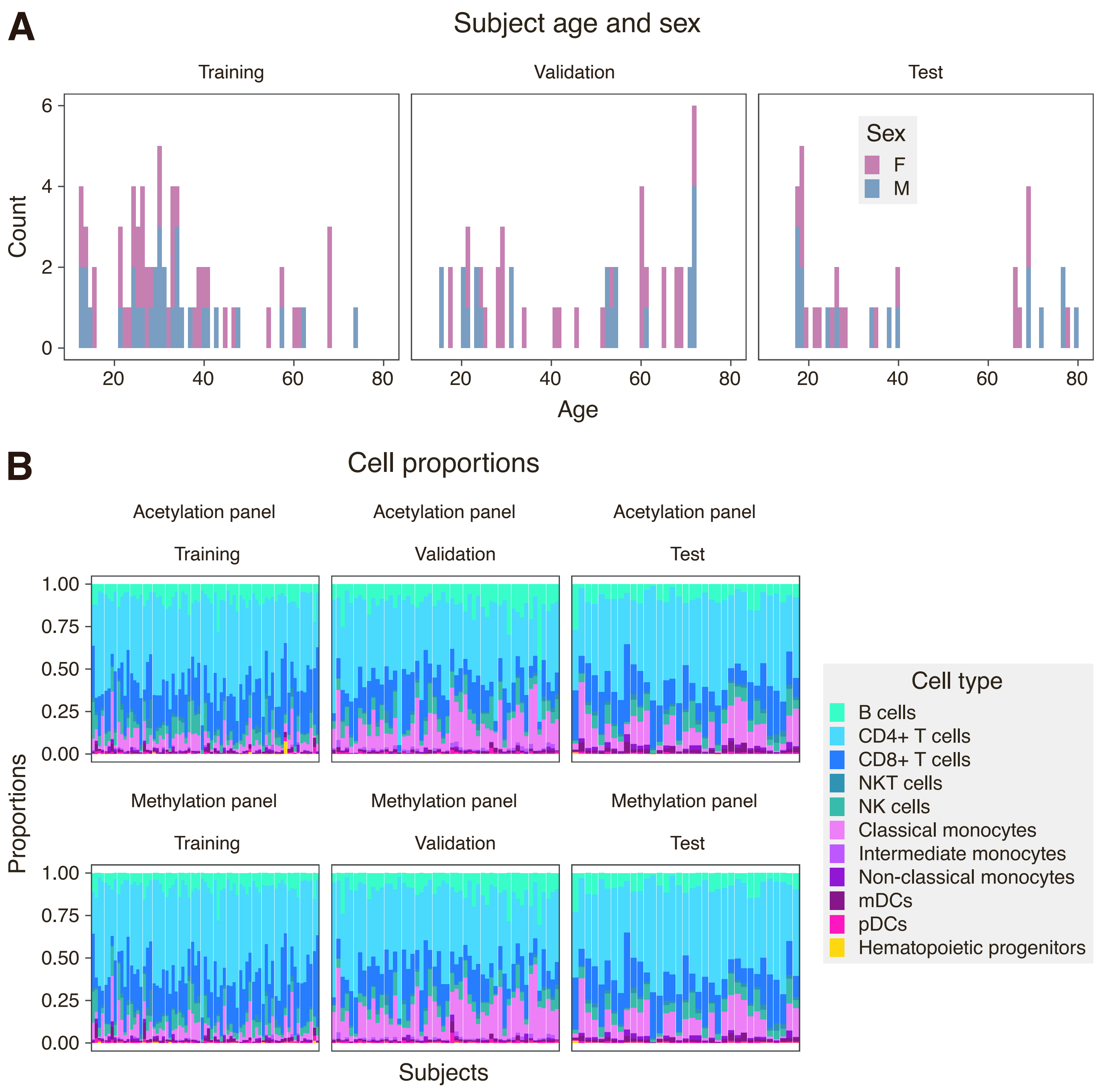

Figure S1. **Summary of EpiTOF data. A)** Distribution of age and sex in training, validation, and test sets. **B)** Distribution of cell proportions for the 11 immune cell sub-types. Each bar corresponds to a subject. Related to **Figure 1**.

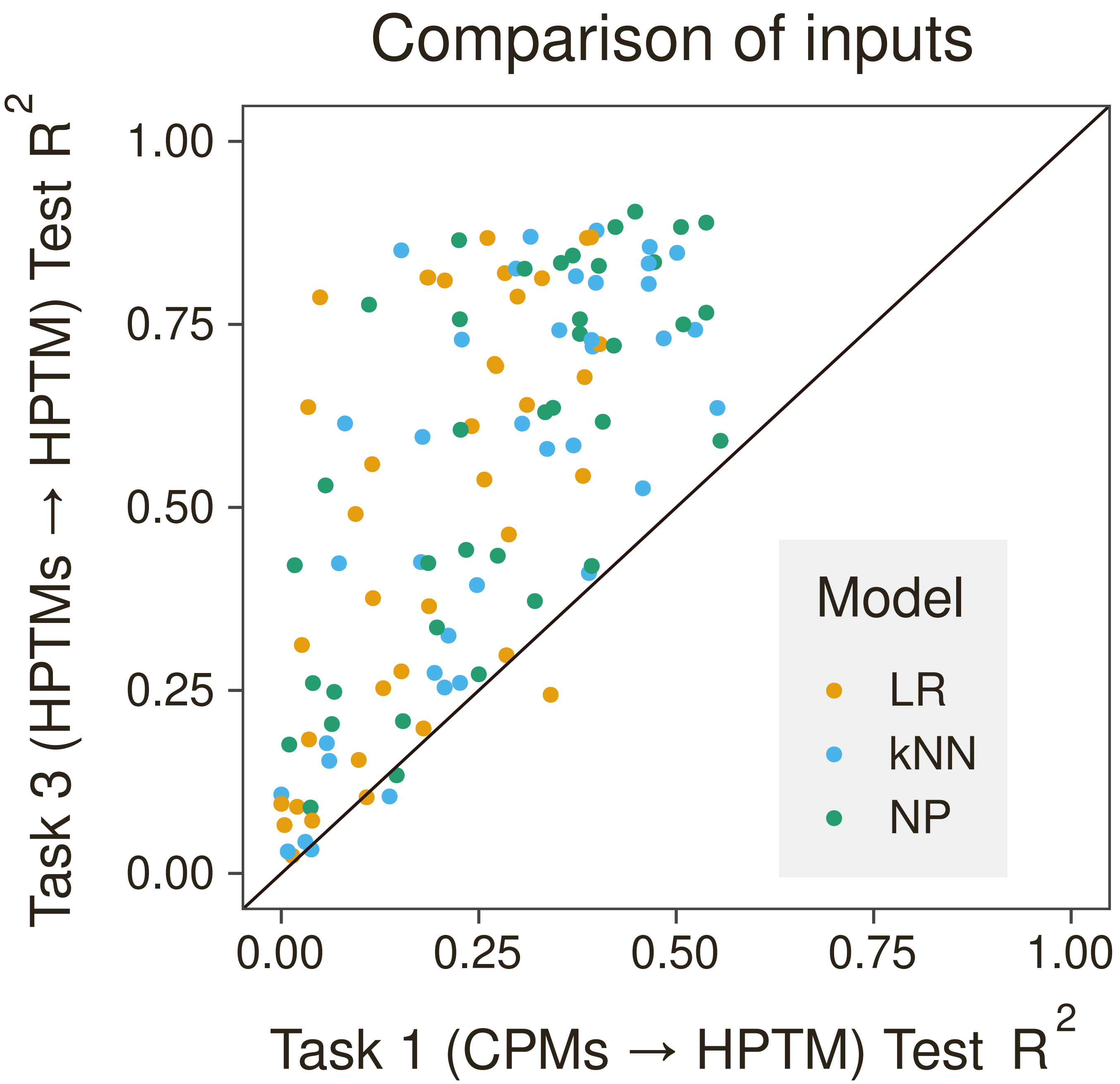

Figure S2. Imputing an HTPM using other HPTMs has higher accuracy than using CPMs. Each dot represents a unique combination of HPTM and algorithm. Related to **Figure 1**.

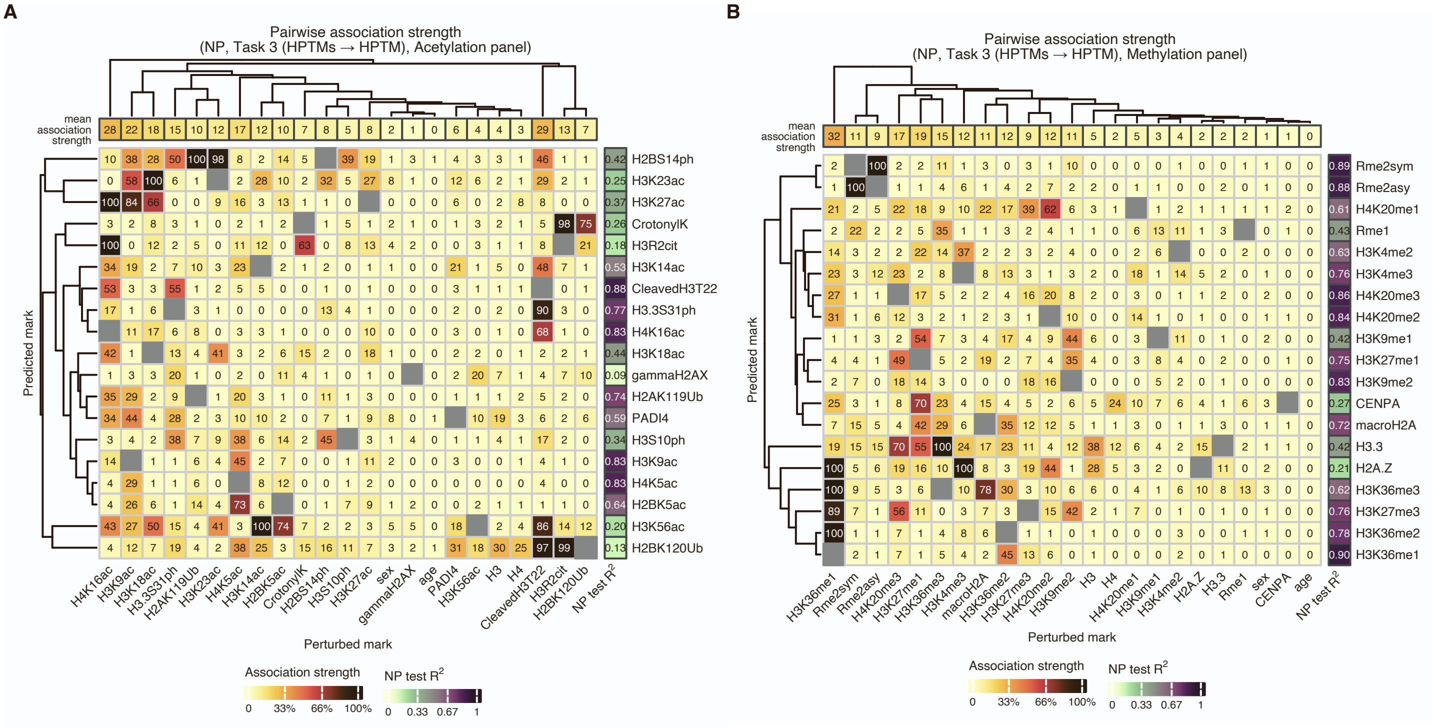

Figure S3. Inferred strength of associations between each pair of HPTM using NP-based models. NP-inferred association strength in test set for pairs of HPTM in **A)** acetylation and **B)** methylation panel. Related to **Figure 2**.

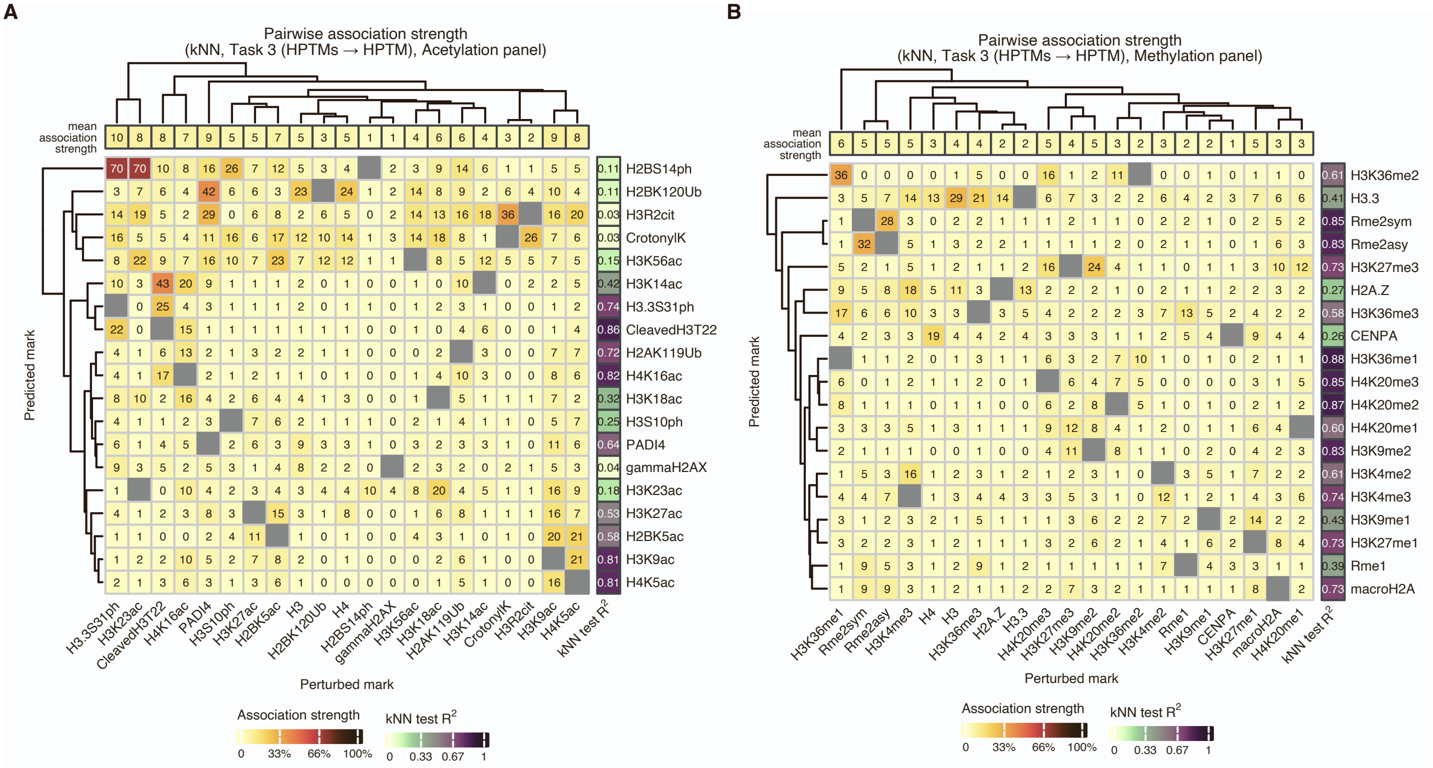

Figure S4. Inferred strength of associations between each pair of HPTM using *k*NN-based models. *k*NN-inferred association strength in test set for pairs of HPTM in **A)** acetylation and **B)** methylation panel. Related to **Figure 2**.

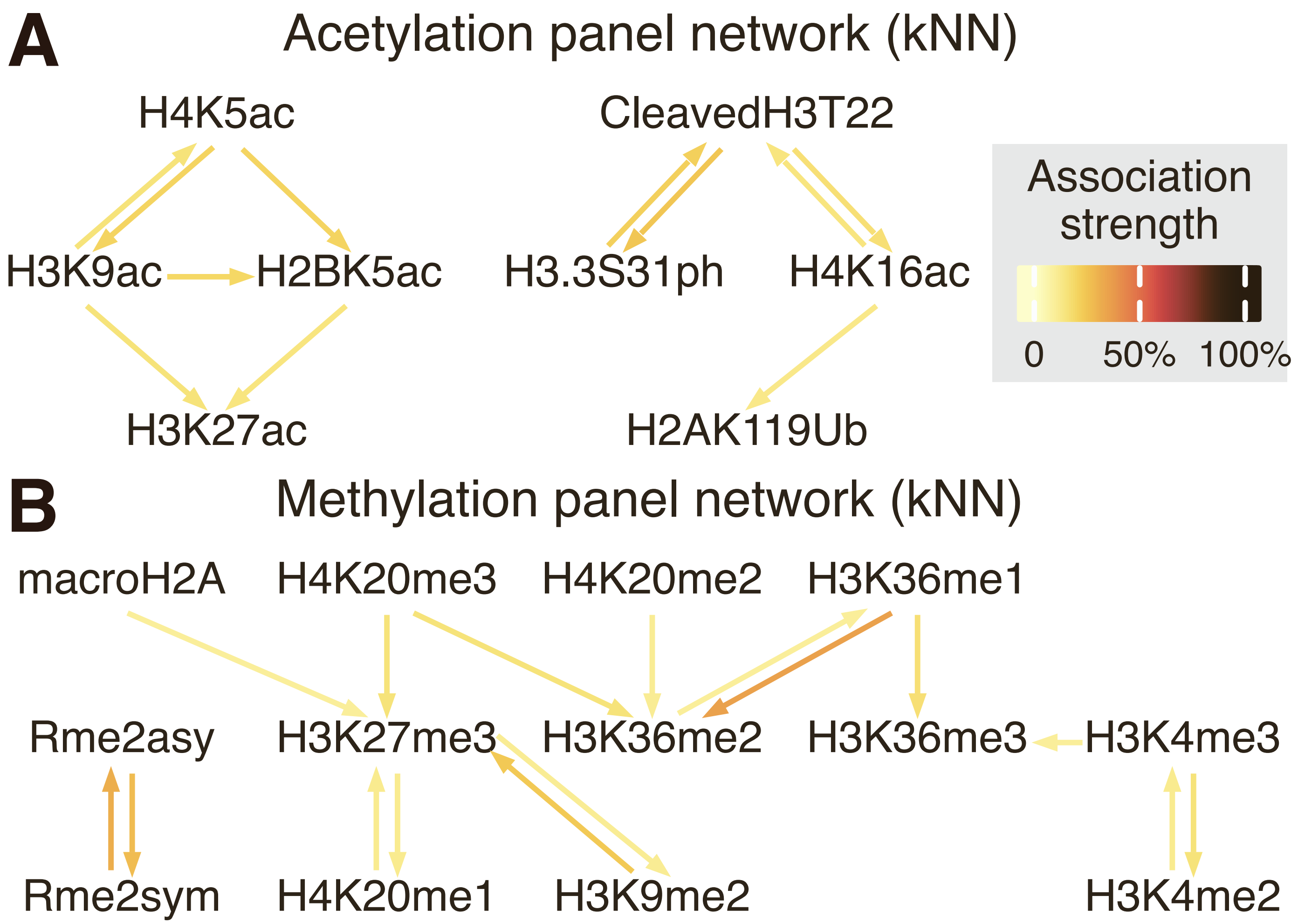

Figure S5. **HPTM association network inferred using *k*NN-based models.** *k*NN-inferred association network in test set for HPTMs in **A)** acetylation and **B)** methylation panel. Related to **Figure 2**.

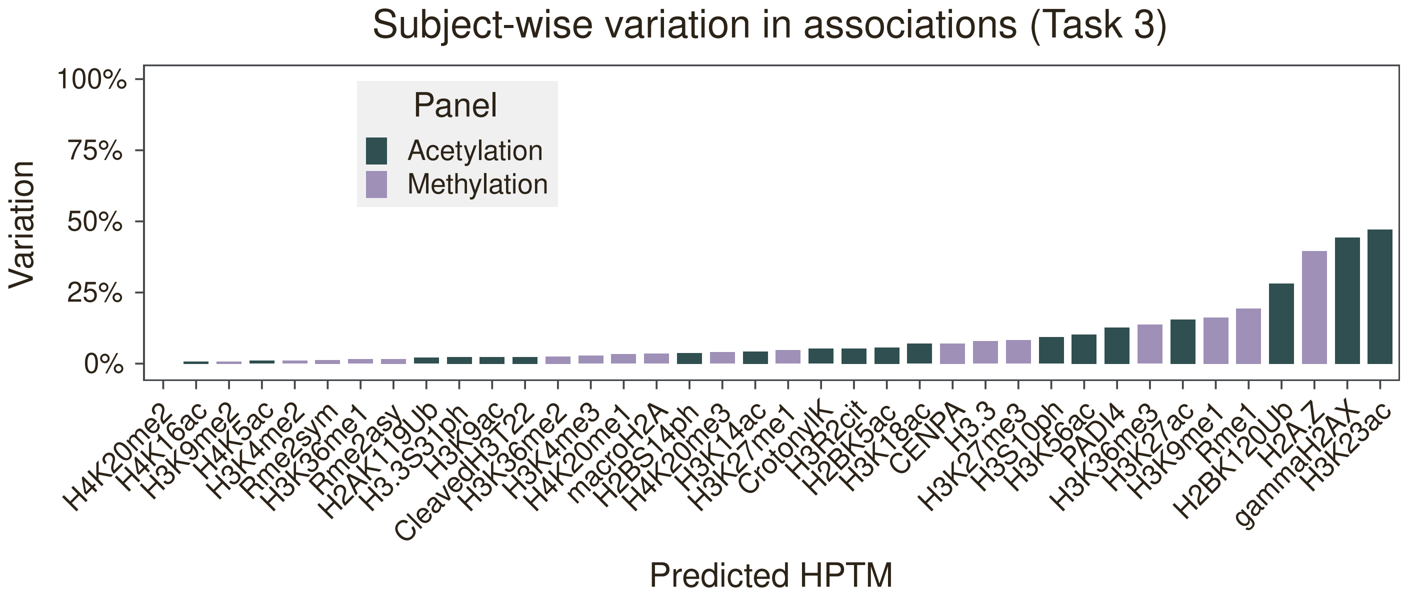

Figure S6. Subject-wise variation in association for each NP-based model when inferring an HPTM using other HPTMs. Related to **Figure 2**.

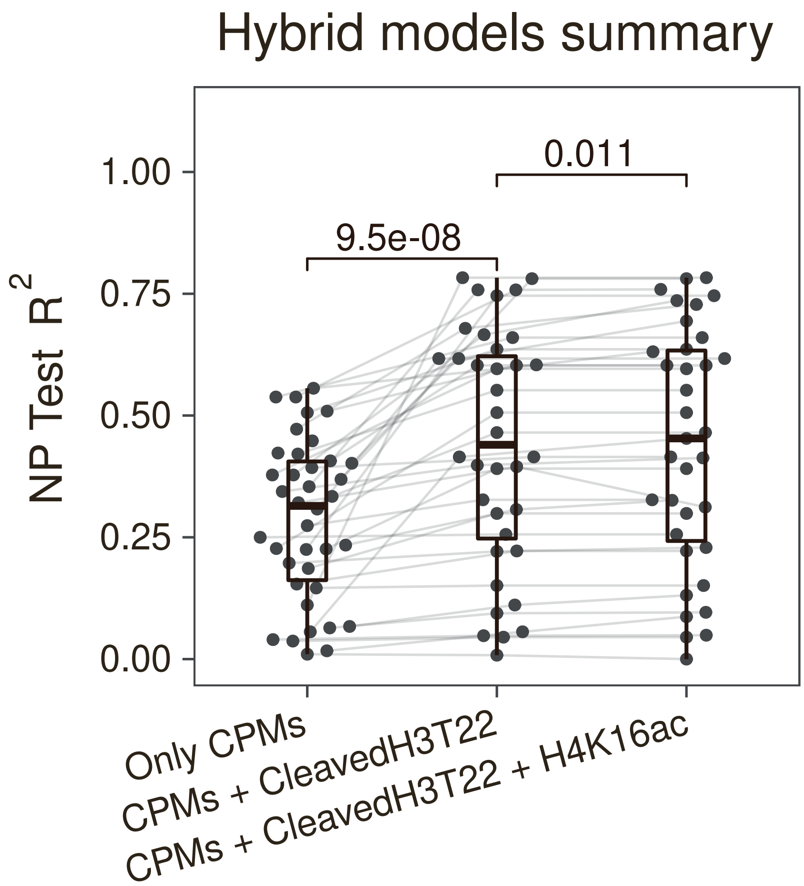

Figure S7. Hybrid NP-based models using CPMs and highly predictive HPTMs (CleavedH3T22, H4K16ac) have significantly higher accuracy than using only CPMs. Each dot corresponds to an HPTM. One-sided, paired Wilcoxon test was used to compute significance of the improvement in R^2 (n = 35 per task). Related to **Figure 3**.

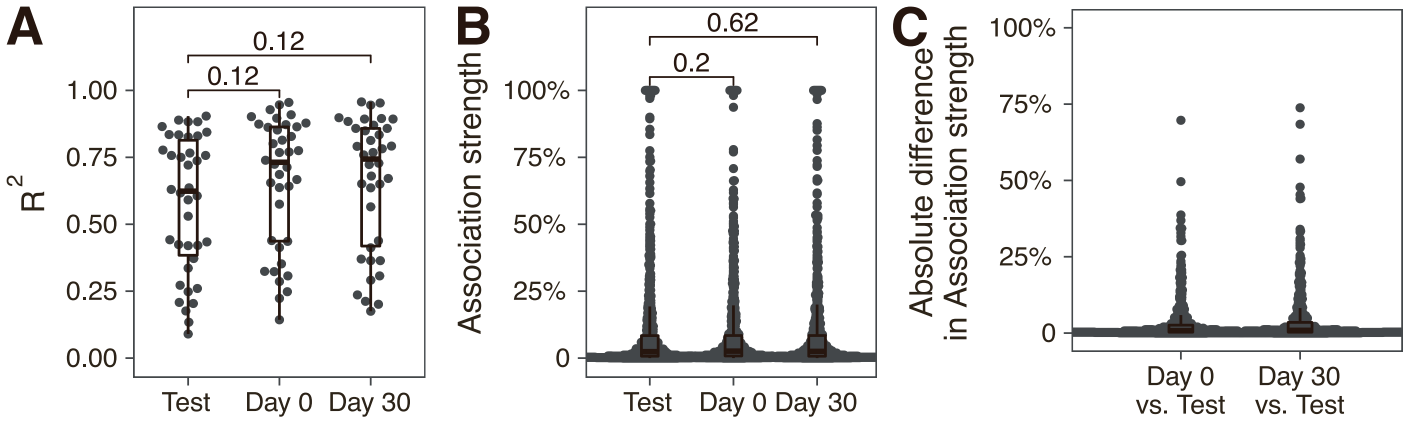

Figure S8. Summary of NP-based models and inferred associations in Influenza vaccine cohort. A) Comparison of R^2 between test set, *Day 0*, and *Day 30*. Each dot represents an HPTM. FDR-adjusted Wilcoxon test was used to compute significance of differences in proportion means across groups (n = 38 per group). **B)** Comparison of association strength between test set, *Day 0*, and *Day 30*. Each dot represents a unique pair of HPTMs. FDR-adjusted Wilcoxon test was used to compute significance of differences in proportion means across groups (n = 684 per group). **C)** Absolute difference in association strength between test set *vs*. *Day 0* and *Day 30* time points. Each dot represents a unique pair of HPTMs. Related to **Figure 4**.

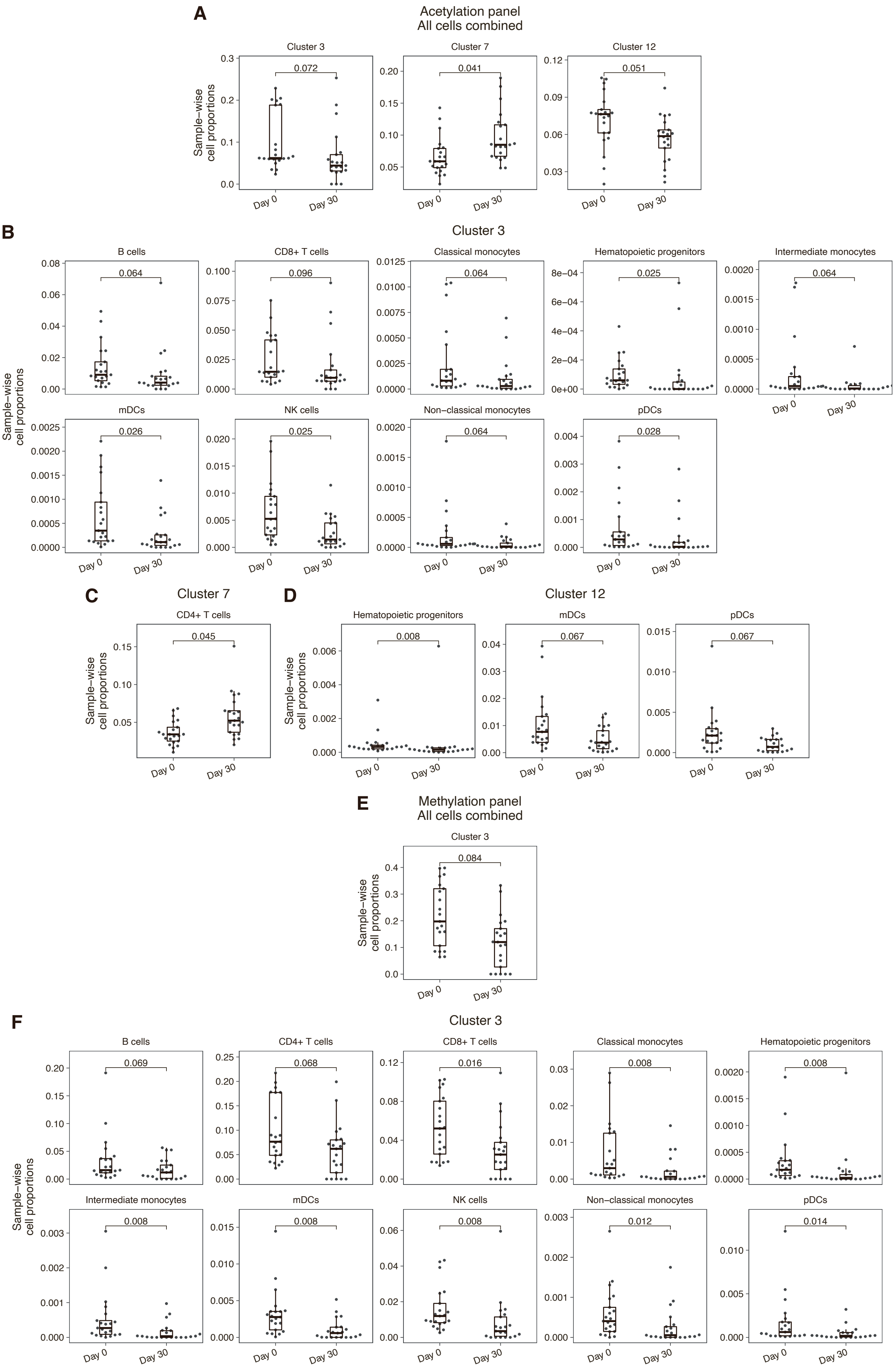

A
Acetylation panel
All cells combined
Cluster 3
0.072
Cluster 7
0.041
Cluster 12
0.051
Sample−wise
cell proportions
Day 0
Day 30
B
Cluster 3
B cells
0.064
CD8+ T cells
0.096
Classical monocytes
0.064
Hematopoietic progenitors
0.025
Intermediate monocytes
0.064
mDCs
0.026
NK cells
0.025
Non−classical monocytes
0.064
pDCs
0.028
C
Cluster 7
CD4+ T cells
0.045
D
Cluster 12
Hematopoietic progenitors
0.008
mDCs
0.067
pDCs
0.067
E
Methylation panel
All cells combined
Cluster 3
0.084
F
Cluster 3
B cells
0.069
CD4+ T cells
0.068
CD8+ T cells
0.016
Classical monocytes
0.008
Hematopoietic progenitors
0.008
Intermediate monocytes
0.008
mDCs
0.008
NK cells
0.008
Non−classical monocytes
0.012
pDCs
0.014

Figure S9. **Differences in cell proportions in HPTM-defined cell clusters 30 days after influenza vaccine.** Sample-wise cell proportions in A) acetylation panel, all cell immune cell sub-types combined, B) acetylation panel, cluster *3*, by immune cell sub-type, C) acetylation panel, cluster *7*, by immune cell sub-type, D) acetylation panel, cluster *12*, by immune cell sub-type, E) methylation panel, all immune cell sub-types combined, and F) methylation panel, cluster *3*, by immune cell sub-type. Each dot represents a subject. All cells from all samples were used to calculate the proportions. We used FDR-adjusted Wilcoxon test to compute significance of differences in proportion means across groups (n = 21 per time point). Related to **Figure 4**.

Table S1: CPMs and HPTMs measured in the EpiTOF data. Related to Figure 1.

Shared between both panels	Acetylation Panel	Methylation Panel
CD11c	CleavedH3T22	CENPA
CD123	CrotonylK	H2A.Z
CD14	gammaH2AX	H3.3
CD16	H2AK119Ub	H3K27me1
CD19	H2BK120Ub	H3K27me3
CD3	H2BK5ac	H3K36me1
CD4	H2BS14ph	H3K36me2
CD45	H3.3S31ph	H3K36me3
CD56	H3K14ac	H3K4me2
CD8	H3K18ac	H3K4me3
HLADR	H3K23ac	H3K9me1
H3	H3K27ac	H3K9me2
H4	H3K56ac	H4K20me1
	H3K9ac	H4K20me2
	H3R2cit	H4K20me3
	H3S10ph	macroH2A
	H4K16ac	Rme1
	H4K5ac	Rme2asy
	PADI4	Rme2sym

Table S2: Summary of EpiTOF experiments. Related to Figure 1

Set	Experiment	Num. of Subjects
Train	Atlanta cohort 1	4
	Atlanta cohort 2	4
	Atlanta cohort 3	4
	Atlanta cohort 4	4
	Atlanta cohort 5	4
	Atlanta cohort 6	4
	Atlanta cohort 7	3
	Atlanta cohort 8	4
	Atlanta cohort 9	4
	Stanford cohort 1	6
	Stanford cohort 2	3
	Stanford cohort 3	7
	South Africa cohort	10
	Oklahoma cohort 1	5
	Oklahoma cohort 2	5
Valid	Twins cohort 1	20
	Twins cohort 2	20
	Atlanta cohort 10	4
	Oklahoma cohort 3	4
	Oklahoma cohort 4	4
Test	BR cohort 1	12
	BR cohort 2	12
	Atlanta cohort 11	4
	Atlanta cohort 12	3
	Atlanta cohort 13	4
Flu vaccine cohort	Atlanta cohort 1	2
	Atlanta cohort 2	1
	Atlanta cohort 3	2
	Atlanta cohort 4	4
	Atlanta cohort 5	4
	Atlanta cohort 6	2
	Atlanta cohort 12	2
	Atlanta cohort 13	4

Table S3: Neural Processes architecture. Related to Figure 1

**Task**	**Encoder dimensions**				**Imputer dimensions**			
	**Input**	**Hidden 1**	**Hidden 2**	**Output**	**Input**	**Hidden 1**	**Hidden 2**	**Output**
Task 1 (CPMs -> HPTM)	16	256	256	512	527	256	256	1
Task 2 (HPTMs -> CPM)	24	256	256	512	535	256	256	1
Task 3 (HPTMs -> HPTM	23	256	256	512	534	256	256	1
Hybrid models	17	256	256	512	528	256	256	1
